# Insight to motor clutch model for sensing of ECM residual strain

**DOI:** 10.1016/j.mbm.2023.100025

**Published:** 2023-10-12

**Authors:** Valeria Panzetta, Claudia De Clemente, Michele Russo, Sabato Fusco, Paolo A. Netti

**Affiliations:** a Department of Chemical, Materials and Production Engineering, University of Naples Federico II, 80125, Naples, Italy; b Centro di Ricerca Interdipartimentale sui Biomateriali, University of Naples Federico II, 80125, Naples, Italy; c Istituto Italiano di Tecnologia, IIT@CRIB, 80126, Naples, Italy; d Department of Industrial Engineering, University of Naples Federico II, 80125, Naples, Italy; e Department of Medicine and Health Sciences “V. Tiberio”, University of Molise, 86100, Campobasso, Italy

**Keywords:** Focal adhesions, Motor clutch model, Residual stress, Elastic strain energy

## Abstract

The mechanical microenvironment strongly affects cell state and decisions. Cell mechanosensing has been described by a *molecular clutch* which gets progressively engaged depending upon the stiffness of the extracellular material. Through the actuation of pulling forces exerted by actin fibres on the mechanosensitive talin-integrin molecular complex, cells sense and react to the stiffness of their surroundings. However, whether the truly cell mechanosensing is regulated by the pure elastic stiffness or by the strain energy density of the ECM is still debated. Here we report that the cell response to change of strain energy density out of loading induced deformation (purely elastic) can be accounted for by including, within the same frame of the molecular clutch model, the residual strain/stress to which the ECM could be subjected before establishing any interaction with the molecular clutches. To include the contribution of residual stresses, an additional spring orthogonal to the ones already present in the original clutch model has been introduced; this spring takes memory of the ECM strain energy when axially deformed before any interaction with cell molecular clutches can occur. To evaluate the influence of strain on the optimum number of clutches, the model has been implemented with different levels of strain. Results suggest that cells undergo a reinforcement process, stiffening the cytoskeleton in response to the ECM stress/strain energy.

## Graphical abstract

**Figure:**
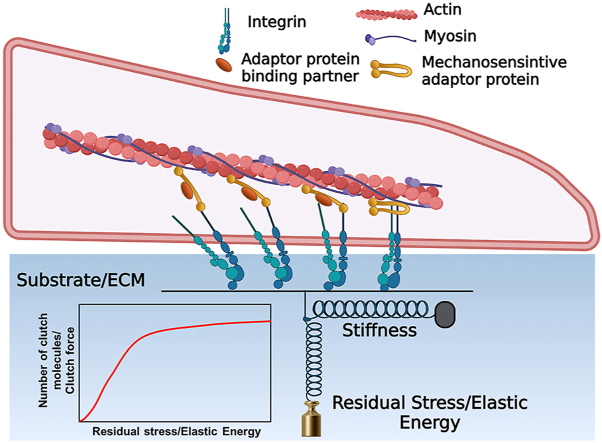

## 1 Introduction

Cells in living tissues are continuously exposed to a plethora of biophysical stimuli coming from their microenvironment, i.e., cells [[1], [2], [3]] and the extracellular matrix (ECM) [[4], [5], [6]]. A growing body of evidence indicates the importance of the mechanical microenvironment in modulating and guiding cell behaviour in both physiological and pathological conditions [[7], [8], [9]]. In this context, ECM mechanics has emerged as a key regulator of cellular fate. Indeed, strong evidence indicates that ECM elasticity plays an important role in regulating cell adhesion and cytoskeleton organisation that ultimately affect proliferation, migration, self-renewal and differentiation [8,[10], [11], [12]]. It is important to note that most of the research exploring the role of ECM elasticity on cell behaviour, has been performed in 2D contexts. The comprehension of its importance in three dimensions seems challenging since it is difficult to independently control matrix stiffness and mesh-size, two parameters that, in 3D matrix, affect cell adhesion and spreading in opposite manner [13]. However, Yuan et al. have recently reported that a local stiffening of 3D hydrogel network produces adhesion, spreading and osteogenic differentiation of stem cells as observed on 2D stiff substrates [10,14]. In recent years, numerous works have also highlighted the important role of time dependent mechanical features of the microenvironment, i.e., viscoelasticity, on cell mechanosensing and mechanotransduction processes. ECM viscoelastic moduli have proved to be effective in regulating cell adhesion, spreading, proliferation, and differentiation [[15], [16], [17], [18], [19], [20]]. The role of the ECM dynamic behaviour was also investigated by considering the effect of ligand mobility (i.e., surface viscosity) on cell adhesion and spreading [21]. Beside ECM elastic and viscoelastic moduli, also the strain energy accumulated within the ECM plays an important role in cell and tissues physiological and pathological conditions. For instance, residual stresses, defined as stresses persisting in the tissue solid phase when all the external loads are removed, have been demonstrated as key regulators in physiology of tissues as veins and arteries [[22], [23], [24], [25]] as well as in solid tumours [[26], [27], [28], [29]]. Residual stresses, generally originated by a non-isotropic tissue growth and/or remodelling [30,31], are important features that together with ECM elastic and viscoelastic moduli uniquely define the mechanical identity of cellular microenvironment. However, most of the literature studies aimed at elucidating the basic mechanism underlying the mechanoregulation in tissue morphogenesis and histogenesis are limited to the role of elastic modulus of the ECM, neglecting or downplaying the important role of residual stresses.

It is widely accepted that the ability of cells to recognize and respond to their mechanical microenvironment resides in a sophisticated adhesion-dependent mechanosensing process, based on the dynamics of macromolecular complexes known as focal adhesions (FAs). FAs are multiprotein complexes that form a dynamic interface between the cell cytoskeleton (CSK) and the ECM [32,33]. When the connection between ECM, FAs and CSK is established, FAs act as a mechanosensing hub allowing the mechanical continuity between cell-inborne cytoskeleton forces and the microenvironmental generated forces. For this reason, they are central in the mechanotransduction and mechanosensing process and hence in tissue remodelling, development, and diseases.

The mechanism governing the dynamics of formation of FAs and its dependence on the extracellular mechanical elastic and viscoelastic microenvironment has been successfully described through the molecular clutch model that connects the force transmission from FAs (referred to as ‘clutches’) to ECM in function of matrix rigidities [[34], [35], [36]], viscoelasticity [20,37] and ligand mobility [21]. Clutch model has been proved suitable to interpret the mechanical coupling between microenvironmental and cell in-borne forces [38]. It was conceived as a stochastic approach to model the dynamics of individual molecular clutches and then extended to describe cell–scale processes like cell migration [34]. Then, it has was used to explain the mechanism by which cells sense the ECM stiffness [34,36] through the action of molecular clutches (constituted by integrins and adaptor proteins), that, connecting the ECM to the cell cytoskeleton, convert the contractile forces generated by myosin molecules into traction forces against the ECM. The constant process of actin polymerization, together with the myosin-generated contractility, power a continuous flow of actin, termed retrograde flow that orients from the leading-edge membrane towards the cell centre. When the clutches are engaged, myosin contractility starts to exert a force against the ECM through to the different bound clutches, progressively slowing down the retrograde flow. The velocity at which this force builds up - the force loading rate - is directly regulated by the ECM stiffness. On very stiff substrates, the force builds up very fast, destabilising the bond and disengaging all the clutches before others can bind. On very soft substrates, the velocity is too low, so that clutches disengage before high forces are reached. Generally, among these two extreme cases, the process is interrupted when the force increases and destabilises the bond. This biphasic behaviour predicts an optimal value for the ECM stiffness (or the loading rate) in correspondence of which the force transmitted to the clutches and to ECM is maximum [34,36].

**Fig. 1:**
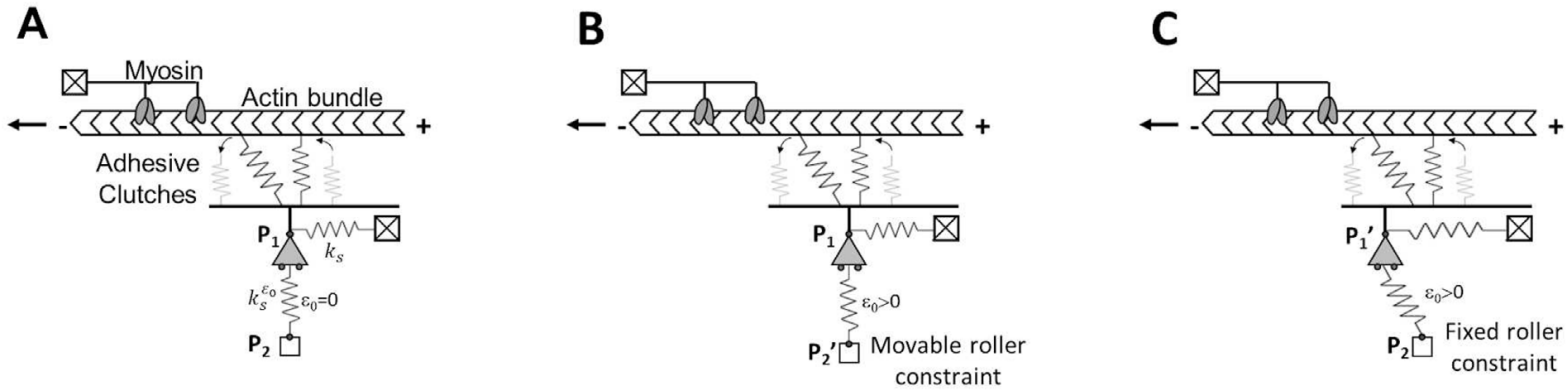
Scheme of the modified clutch model when no residual strain (A), axial strain (B) or transverse strain (C) are introduced in the orthogonal spring $k_{s}^{\varepsilon_{o}}$.

In our recent work [39], we demonstrated that fibroblasts are able to sense the difference in strain energy distribution, and therefore residual stresses, in prestressed substrates by activating a process of cellular reorganisation that leads to an increase in contractility and stiffness of the actin cytoskeleton. We also supported our experimental results by a first modification of the molecular clutch model. Here, we provide an insight to the mechanistic explanation of how FAs sensing is residual stress-regulated by an extension of the clutch model: a stochastic simulation that, including an additional spring in the perpendicular direction ($k_{s}^{\varepsilon_{o}}$ in Fig. 1) with a residual strain along its own direction (or in the transverse one), takes memory of stored residual stresses. For the sake of completeness, to include any solicitations to which the ECM could be subjected, and in particular to the residual strain/stress generated by the interaction of the molecular clutches and the external microenvironment, in the following, we will refer sometimes to the material elastic strain energy (instead of to the residual strain/stresses), intending the stored energy within the material as a function of the total elastic strain. We modelled two different substrate stiffnesses and for each substrate we simulated different levels of residual strain. Simulations indicated that there was a positive relationship between the number of clutches involved in FAs assembly and the elastic strain energy suggesting that the increasing of material strain energy is perceived by the cells as an apparent increase of material stiffness. Therefore, according to experimental observation, at increasing level of strain energy cells undergo a reinforcement process of their in-borne cytoskeleton forces leading to a new mechanical state as they were cultured on a stiffer material.

This study supplies more insight to the discussion of the importance of the molecular clutch model in predicting the implications of solid stresses in both tissue physiology and pathology.

## 2 Materials and methods

### 2.1 Equations governing motor clutch model

The motor clutch model, as schematized in Fig. 1, was implemented to study the role of residual strain in cell mechanosensing. Specifically, we introduced a modification to the classical clutch model [35] by including a second spring in the perpendicular direction ($k_{s}^{\varepsilon_{o}}$ in Fig. 1). The spring $k_{s}^{\varepsilon_{o}}$ is connected by a pin-roller constraint at **P**_**1**_ to the spring representing the substrate and already present in the canonical clutch model ($k_{s}$ in Fig. 1) and by a roller constraint at **P**_**2**_. It is able to take memory of residual strain $\varepsilon_{0}$ that can be introduced axially by moving the roller constraint from **P**_**2**_ in the position **P**_**2**_**’** or transversally by moving the pin-roller from **P**_**1**_ to **P**_**1**_**’**. The residual strain is defined as:

(1) $$\varepsilon_{0}=\frac{l^{\prime }-l}{l}$$

where l and $l^{\prime }$ are, respectively, the undeformed and deformed length of the spring $k_{s}^{\varepsilon_{o}}$, the first one given by the distance between **P**_**1**_ and **P**_**2**_ and the second one by the distance between **P**_**1**_ and **P**_**2**_**’** in the case of the axial deformation or by the distance between **P**_**1**_**’** and **P**_**2**_ in the case of the transverse deformation.

The master equations governing the dynamic evolution of the system, in terms of clutch engaging and disengaging events, are those introduced by Bengasser et al. with some modifications that consider the presence of the spring $k_{s}^{\varepsilon_{o}}$ and the residual strain $\varepsilon_{0}$.

The change of probability that the ith-clutch is engaged is given by

(2) $$\frac{dp_{b,i}}{dt}=\left(1-p_{b,i}\right)k_{on}-p_{b,i}k_{off,i}^{*}$$

$p_{b,i}$ and $\left(1-p_{b,i}\right)$ are respectively the probability of a clutch to be engaged or disengaged, whereas $k_{on}$ and $k_{off,i}^{*}$ are the association and the dissociation rate for the molecular clutch. $k_{on}$ is constant and $k_{off,i}^{*}$ is force-dependent through the Bell equation

(3) $$k_{off,i}^{*}=k_{off}e^{\frac{F_{c,i}}{F_{b}}}$$

where $k_{off}$ is the clutch unloaded off rate, $F_{c,i}$ the force generated inside the clutch and $F_{b}$ the characteristic rupture force. The clutch force $F_{c,i}$ follows the Hooke's Law, relating the extension of the clutches through the clutch spring constant $k_{c}$, assumed equal for all clutches:

(4) $$F_{c,i}=k_{c}*\left(x_{c,i}-x_{s}\right)$$

where $x_{c,i}$ and $x_{s}$ are respectively the ith-clutch and substrate position. By introducing the pin-roller constraint at **P**_**1**_, the model is formulated in one spatial dimension for the clutches and, consequently, in the Eq. (4) they are all axially oriented and cannot rotate or bend by contributing to the equilibrium of the vertical force. This simplification refers to cell deformations at the level of the adhesion plane (only tangential) though, in principle, the model can be generalised to two and three dimensions and vertical contributions coming from the clutches can be included. The relationship between them can be derived by the equilibrium equation between the overall clutch force, that is the sum of forces generated in all engaged clutches, the substrate force, that is the sum of the forces generated in $k_{s}$ and $k_{s}^{\varepsilon_{o}}$, and the force associated to the residual strain $\varepsilon_{0}$, named in the following $F_{0}$. In particular, when the force $F_{0}$ is applied axially, it is completely balanced by the reaction constraints and the equilibrium equation is simplified in the following

(5) $$\sum_{i}F_{c,i}=F_{s}$$

where the summation is referred only to the engaged clutches and the substrate force is

(6) $$F_{s}=k_{s}^{\varepsilon_{o}}\left(\sqrt{{l^{\prime }}^{2}+x_{s}^{2}}-l\right)\frac{x_{s}}{\sqrt{{l^{\prime }}^{2}+x_{s}^{2}}}+k_{s}x_{s}$$

when the force $F_{0}$ is applied transversally, the equilibrium equation is

(7) $$F_{0}+\sum_{i}F_{c,i}=F_{s}$$

where:

(8) $$F_{0}=k_{s}^{\varepsilon_{o}}\varepsilon_{0}l+k_{s}x_{0}$$

and, based on simple geometric considerations, it is possible to derive the relationship linking $x_{0}$ and $\varepsilon_{0}$.

(9) $$\varepsilon_{0}=\frac{\sqrt{l^{2}+x_{0}^{2}}}{l}-1$$

The F-actin retrograde velocity is related to the force $F_{s}$ by the Hill's equation

(10) $$v_{f}=v_{u}\left(1-\frac{F_{s}}{n_{m}F_{m}}\right)$$

where $v_{u}$ is the velocity of free myosin motors, $n_{m}$ is the number of myosin motors and $F_{m}$ is the stall force for the single myosin motor.

The last equation defines the velocity for the ith-clutch as a weighted average of the velocity of engaged and disengaged clutches, the first one equal to the velocity of the substrate and the second one to that of F-actin

(11) $$\frac{dx_{c,i}}{dt}=\left(1-p_{b,i}\right)\frac{dx_{s}}{dt}+p_{b,i}v_{f}$$

### 2.2 Simulation algorithm

The system of equations (2, 3, 4, 5/7, 10, 11) has been solved using a Gillespie stochastic algorithm. Specifically, the event times (clutch engaging or disengaging) are randomly generated according to the following equation:

(12) $$t_{\frac{eng}{diseng},i}=-\ln\left({random}_{i}\right)/k_{i}$$

where ${random}_{i}$ is a random number between 0 and 1 and $k_{i}$ is the kinetic rate for a clutch to engage ($k_{on}$) or disengage ($k_{off,i}^{*}$). The engaging and disengaging times for all the clutches are then compared and the minimum time event is evaluated and executed, changing the state of the corresponding clutch from engaged to disengaged or *vice versa*. The time vector is then advanced by the minimum calculated event time.

The engaged clutch positions are also advanced before solving the equilibrium equation, by the product of the F-actin retrograde flow rate and time step and the position of disengaged clutches is set equal to the substrate position.

The number of simulated events has been limited 100 ​k for all analysis and the simulation has been executed for 25 values of n_c_ logarithmically spaced between 10 and 200 for the substrate stiffness equal to 0.1 ​pN/nm and for 20 values of n_c_ logarithmically spaced between 10 and 1000 for the substrate stiffness equal to 1 ​pN/nm. $k_{s}^{\varepsilon_{o}}$ and $k_{s}$ are set equal.

The list of the parameters used in the model is reported in Table 1.

**Table 1:** List of the parameters used in the model.

Parameter	Value
Motor stall force	2 ​pN
Motor unloaded velocity	120 ​nm/s
Clutch bond rupture force	2 ​pN
Clutch on-rate	0.3 s^−1^
Clutch unloaded off-rate	0.1 s^−1^
Clutch spring constant	0.8 ​pN/nm

## 3 Results and discussion

### 3.1 Description of the model

The motor clutch model can explain the mechanism by which cells sense ECM stiffness [34,36]. Through the action of molecular clutches-constituted by integrins and adaptor proteins -that convert the contractile forces generated by myosin molecules into traction forces against the ECM, the model envisages the equilibrium between inside/outside cell forces. These forces acting on molecular clutches reduce the actin retrograde flow, due to the constant process of actin polymerization and depolymerization and increase force loading on the substrate in a stiffness-dependent manner. We hypothesise that an increase of the residual stress within the material and/or substrate strain energy, could be captured by the clutch model by a similar mechanism. Therefore, we modified the clutch model to consider the effect of the elastic strain energy accumulated inside the cell substrate also in presence of residual strain. As illustrated in Fig. 1, the anchorage of molecular clutches to the substrate is modelled as constituted by 2 linear elastic springs arranged orthogonally and connected to each other by a pin-roller constraint at **P**_**1**_.

The spring $k_{s}$, that is the “substrate spring”, can deform when the clutch binding events start to occur, by horizontally translating the pin-roller constraint as happens in the original motor clutch formulation [34,35]. In the following section it will be shown that, thanks to the presence of the spring $k_{s}$, the model retains its ability to consider the effect of substrate stiffness (i.e., modulus) and to provide a full description of stiffness-sensing in cells. However, due to the linear elastic behaviour of the substrate spring, the original clutch model cannot describe the cell's ability to perceive residual strain in addition to substrate stiffness. In fact, when an external force $F_{0}$, responsible for the emergence of the residual strain $\varepsilon_{0}$, is applied to the spring $k_{s}$ (Fig. S1), it is completely balanced by the spring reaction also when the molecular clutches begin to bind the F-actin to the substrate. Therefore, the force transmitted by the molecular clutches to the extracellular environment does not depend on the strain energy accumulated within the ECM, but only on the level of the extra strain imposed on top of the already existing residual strain $\varepsilon_{0}$ in the material (see Fig. S2). To enrich the original formulation of the model, making it able to describe the cell ability to perceive the total elastic strain energy, we introduced a second spring $k_{s}^{\varepsilon_{o}}$, orthogonal to the first one, which accounts for the strain energy accumulated within the substrate, when a given strain $\varepsilon_{0}$ is introduced [39]. Thus, orthogonal spring has been introduced so that it satisfies two main requirements: *i*) to not alter the behaviour of the system in absence of accumulated strain energy; *ii*) if a strain is applied inside the orthogonal spring and along its axial direction (Fig. 1B), it does not modify the initial geometrical configuration of the mechanical system.

### 3.2 Comparison between solutions derived from canonical and modified version of motor clutch model

In order to verify the first requirement, the analysis has concerned a comparison between the original clutch model and its modified version here proposed, to understand if their solutions are different in control condition (when no residual strain is introduced in the orthogonal spring).

**Fig. 2:**
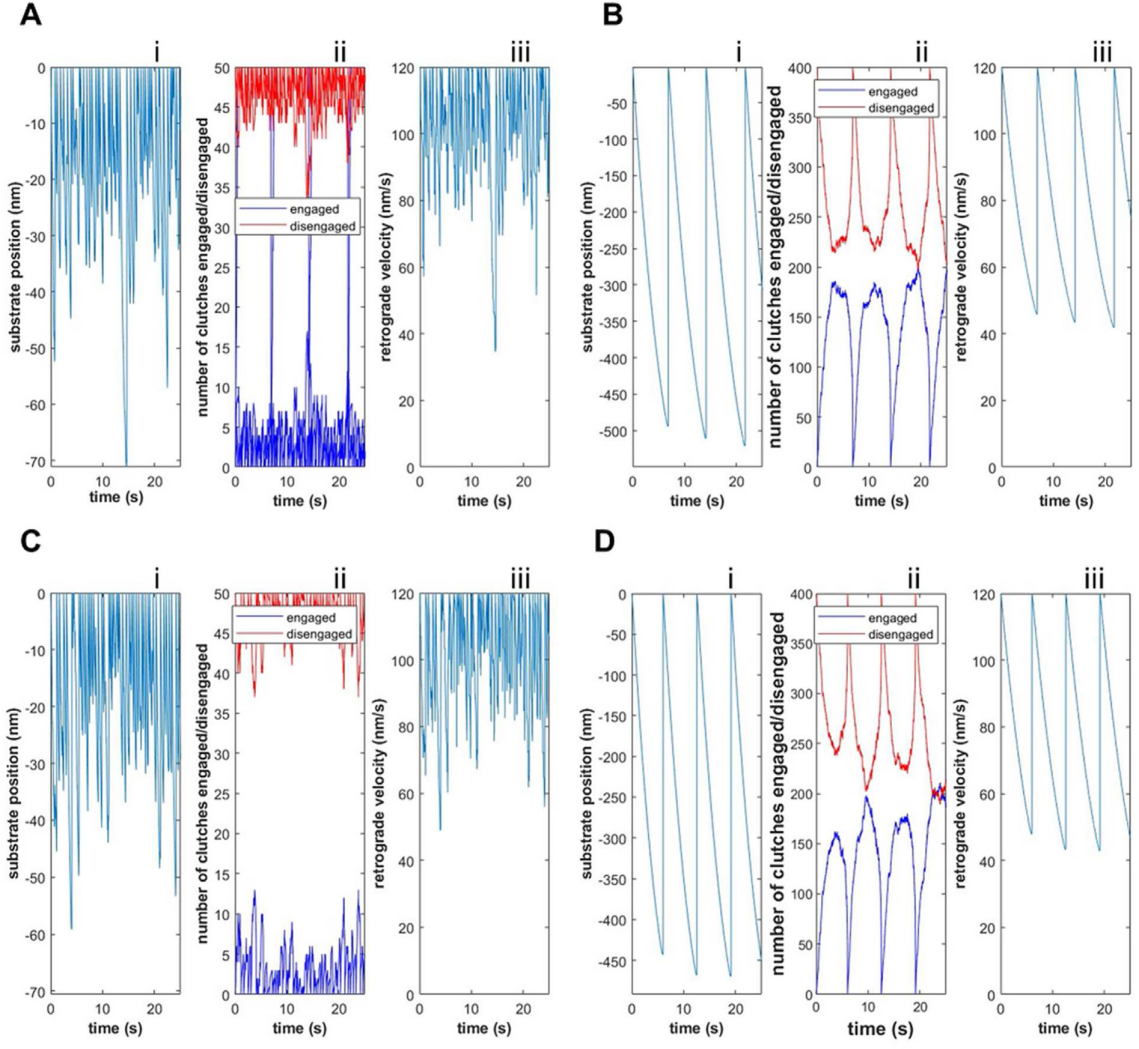
Substrate position (Ai-Di), number of engaged and disengaged clutches (Aii-Dii) and retrograde actin flow rate (Aiii-Diii) as function of time, predicted by the canonical (A–B) and modified version (C, D) of motor clutch model behaviour described with a Monte-Carlo simulation by using the canonical (A–B) and modified version (C–D) of the motor clutch model for two different numbers of available clutches nc, 50 (A–C) and 400 (B,D). k_s ​= ​1 ​pN/nm for all simulations.

As shown in Fig. 2, in both original and modified motor clutch simulations, the clutch engagement operates in two dynamic regimes^33^, the first one called ‘frictional slippage’ (Fig. 2Aii, Cii), and the second one indicated as a ‘load-and-fail’ regime (Fig. 2Bii, Dii), related to the two cases in which a low and high number of clutches, set equal to 50 and 400 respectively, are available inside the system and the substrate stiffness is posed equal to 1 ​pN/nm. When a low number of clutches is introduced, the substrate results to be very stiff: the molecular clutches engage the F-actin bundles, the breaking strength is reached very quickly because of a rapid build-up of myosin contractile tension and an abrupt clutch disengagement is induced. Contrariwise, with a high number of clutches, tension inside engaged ones develops more slowly and the time of interaction between F-actin and engaged clutches, together with their number, increases, allowing the transmission of higher tension to the substrate spring. When the load reaches the limit values, the failure of a single clutch results in the failure of the global system, allowing the substrate to return to its initial position (substrate position equal to zero). At this point the cycle might repeat, inducing system fluctuation over time (Fig. 2B–D).

Furthermore, outputs for both motor clutch models (specifically substrate position (Fig. 2Ai-Di) and retrograde actin flow rate (Fig. 2Aiii-Diii) as function of time) indicate that the dynamics of the system results to not be substantially affected by the introduction of the orthogonal spring in the modified model. To corroborate this statement, average solutions for both models are compared when 100 ​k Monte Carlo runs are performed.

**Fig. 3:**
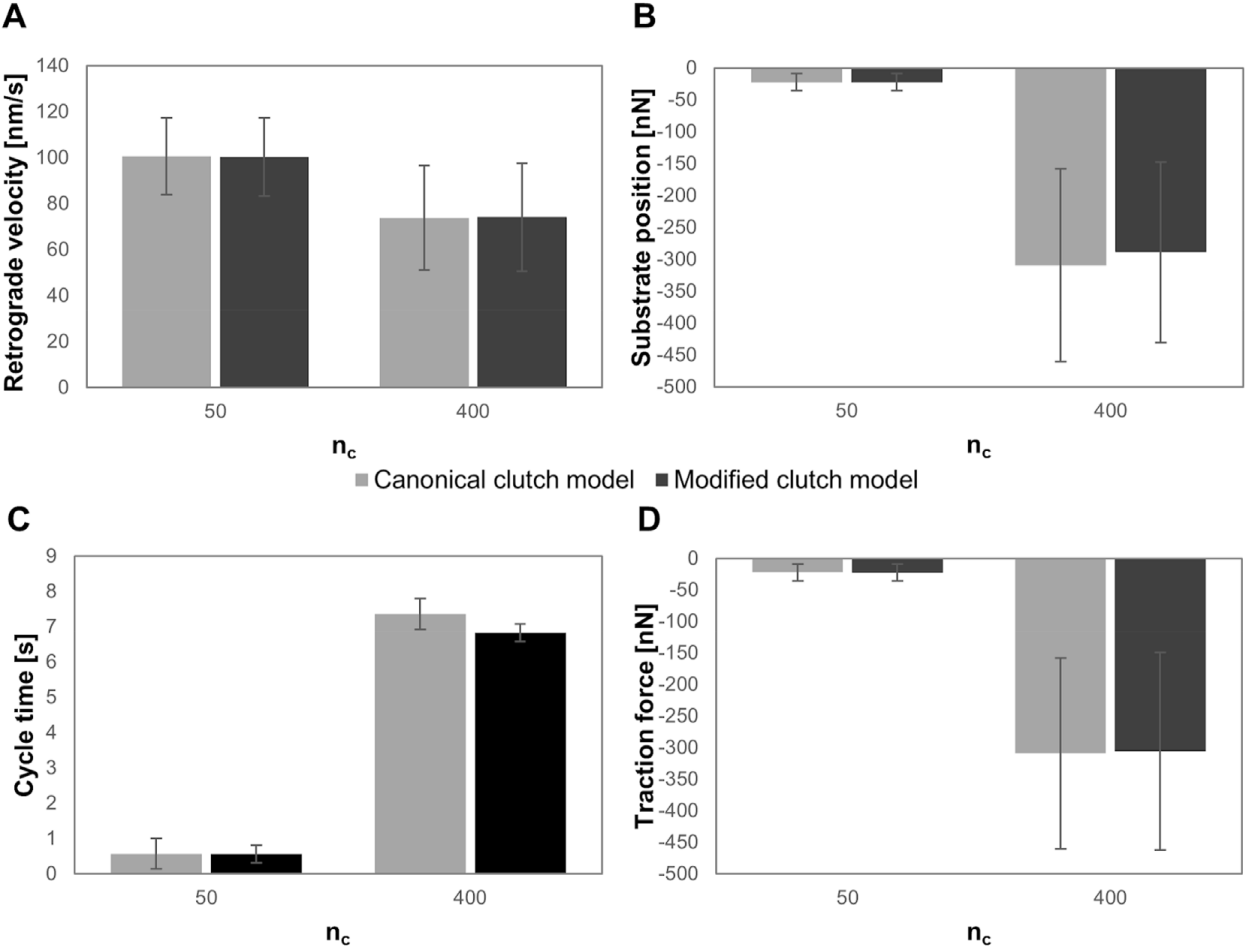
Average values of retrograde actin flow rate (A), substrate position (B), cycle time (C), representing the time occurring between two successive failure events, and traction force (D), predicted by the canonical (light grey bars) and modified version (dark grey bars) of motor clutch model for two different numbers of available clutches n_c_, 50 and 400. $k_{s}=1\frac{pN}{nm}$ for both simulations. Number of Monte Carlo runs equal to 100 ​k.

As shown in Fig. 3, average values of retrograde actin flow rate, substrate position, cycle time (representing the time occurring between two successive failure events) and traction force exerted inside the substrate spring are closely similar, indicating that the modification of the canonical model does not affect substantially the dynamics of the system when no residual strain is introduced inside the orthogonal spring. In addition, the average retrograde actin flow rate has been calculated for different values of the available clutches.

**Fig. 4:**
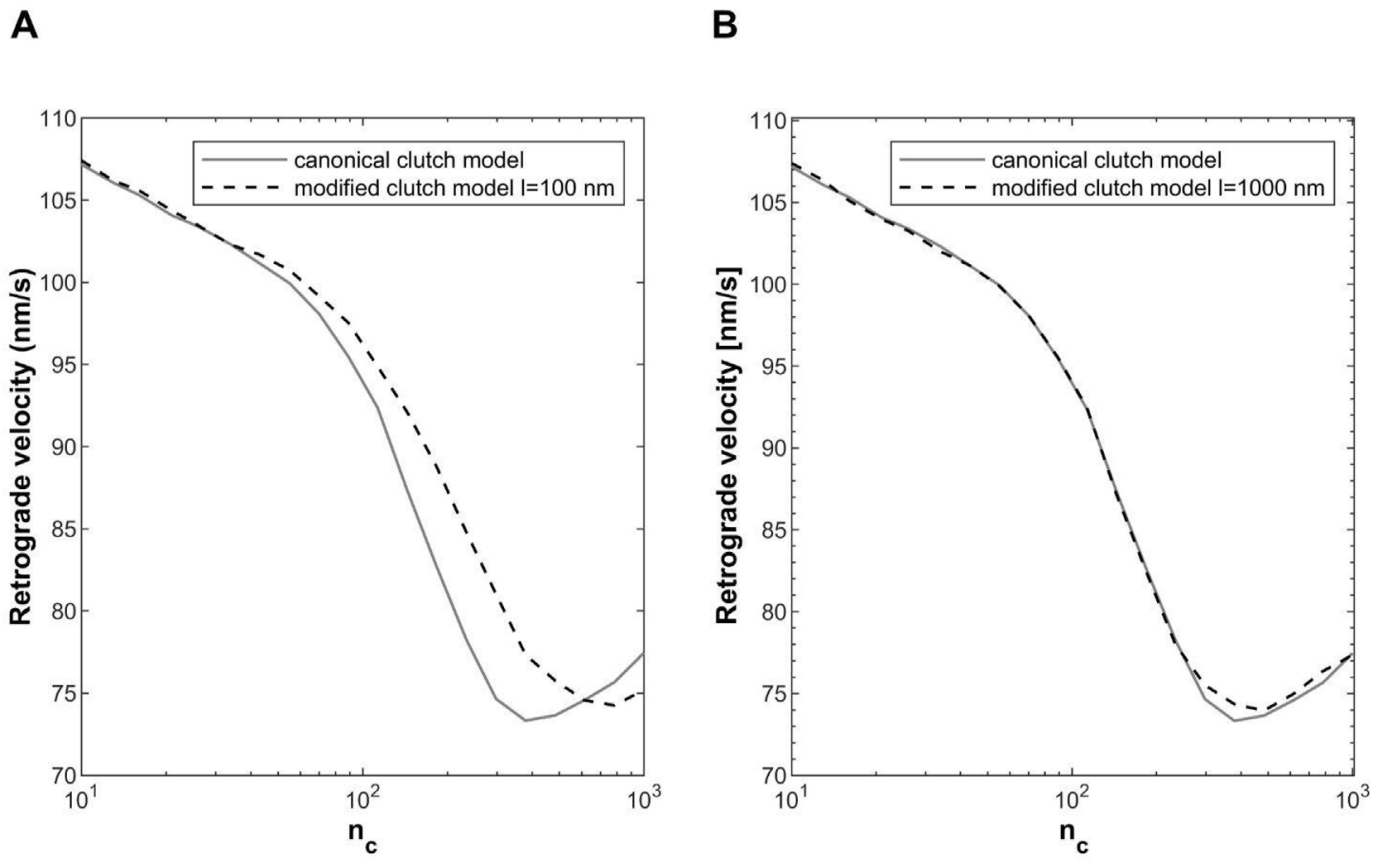
Retrograde actin flow rate as a function of n_c_ predicted by the canonical (light grey solid curve) and modified version (black dashed curve) of motor clutch model with no solid strain. The length of the orthogonal spring was assumed to be equal to 100 ​nm ​(A) and 1000 ​nm ​(B). $k_{s}=1\frac{pN}{nm}$ for both simulations. Number of Monte Carlo runs equal to 100 ​k.

In Fig. 4A, the two curves representing the retrograde actin flow rate as a function of n_c_, are almost overlapping, and the optimum number of clutches, that is the minimum of the curve, is considerably the same for both canonical and modified motor clutch models. Importantly, the minimum of the curve defines the optimum number of the clutches. It is associated as reported for the optimal stiffness (Fig. S2), with the maximal traction force cells are able to exert on the substrate (i.e., maximal adhesion strength) and matches experimental observations reported to date [35,40]. Nevertheless, it should be pointed out that different values for the length of the orthogonal spring have a significant impact on the dynamics of the system. In fact, the reduction in the length of the spring $k_{s}^{\varepsilon_{o}}$ reflects into a stiffening effect of the system, as documented by the shift of the minimum of the retrograde flow velocity towards higher values of n_c_, when the substrate spring stiffness is held constant (Fig. 4B). As a matter of fact, this result indicates that a short orthogonal spring requires a bigger number of clutches to counterbalance the resistance of the substrate (the cell is increasing the adhesion site dimension) and is perceived as an additional stiffness by the model. Dually, this stiffening effect is also reported as a reduction of the optimum stiffness and an increase of the time required for the clutch force to build up and cause the failure of the system^36^. An increase in n_c_ by itself would shift the optimum higher and eventually stall the system^31,32^, but here the motors' number is always set equal to the clutches’ number and the compensatory increase in n_m_ seems to rescue the stalled system. On this basis, the analyses of the residual strain effect have been performed in the successive subsection for a length of the orthogonal spring equal to 1000 ​nm.

### 3.3 Shifting of optimum n_c_ as sensing mechanism of residual strain

In order to evaluate the second requirement and the influence of residual strain - substrate strain energy - on the optimum number of clutches, the proposed model has been implemented by introducing 7 different levels of axial solid strain (0, 0.1, 0.25, 0.5, 1, 2 and 3) and 2 values of substrate stiffness ($k_{s}=1\;\frac{pN}{nm}$ and $0.1\frac{pN}{nm}$).

**Fig. 5:**
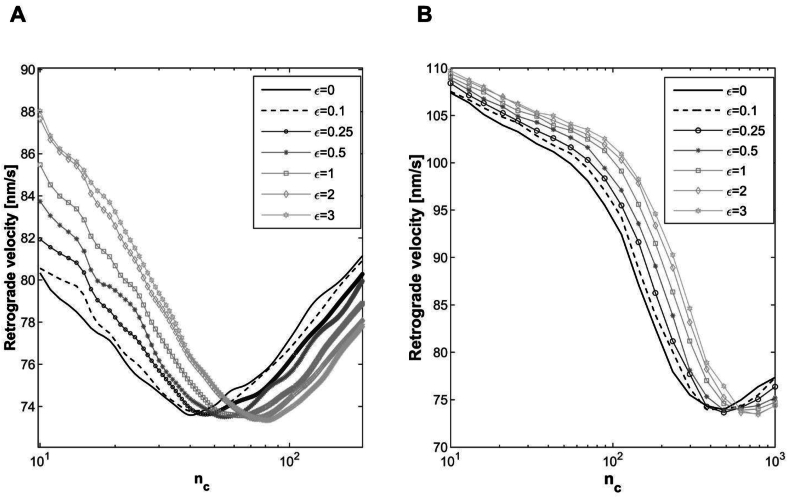
Retrograde actin flow rate as a function of n_c_ predicted by the modified version of motor clutch model when 7 different levels of residual strain (0, 0.1, 0.25, 0.5, 1, 2 and 3) are prescribed to the orthogonal spring. $k_{s}=0.1\frac{pN}{nm}$ (A) and $1\frac{pN}{nm}$ (B). Number of Monte Carlo runs equal to 100 ​k.

Considering that the algorithm is highly time-consuming, the analysis reported in Fig. 5 have been performed for not more than 25 values of n_c_ logarithmically spaced; then the curves have been interpolated with a cubic spline interpolation function to determine the optimum values of clutches for both spring stiffness and for all residual strain considered. As expected, the system continues to manifest a relevant sensitivity to the substrate stiffness and the optimum number of clutches increased by a factor of 10 passing by 40 on the soft substrate ($k_{s}=0.1\;\frac{pN}{nm}$) to 445 on the stiffer one ($k_{s}=1\;\frac{pN}{nm}$). More importantly, as shown in Fig. 5, when the residual strain is applied along the axial direction and strain increases from 0 to 3, the optimum number of clutches shifts towards higher values, indicating a stiffening effect of the system: a greater number of clutches is required to counterbalance the resistance of a pre-strained substrate.

**Fig. 6:**
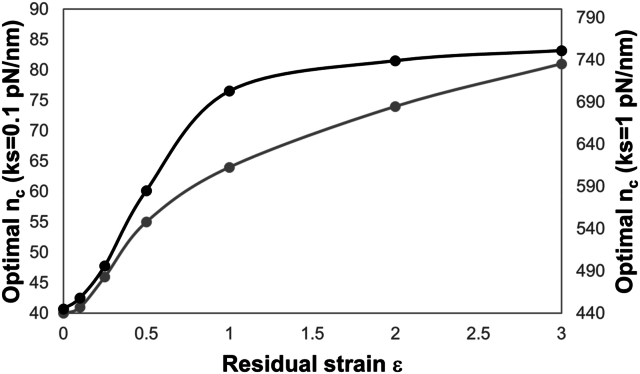
Optimal n_c_ predicted by the modified version of motor clutch model when 7 different levels of residual strain (0, 0.1, 0.25, 0.5, 1, 2 and 31) are prescribed to the orthogonal spring. $k_{s}=0.1\frac{pN}{nm}$ (grey curve) and $1\frac{pN}{nm}$ (black curve).

In particular, the optimal n_c_ exhibits an increase from 3 ​% to 103 ​% on the soft substrate and from 3 ​% to 69 ​% on the stiffer one respect to the control condition (residual strain to 0), when solid strain passing from 0.1 to 3 are axially introduced in the orthogonal spring (Fig. 6). All the reported results refer to simulations where the undeformed/reference length of the orthogonal spring is held constant and equal to 1000 ​nm; this means that the deformed length of that spring increases by enhancing the level of residual strain, passing from 1000 to 4000 ​nm as the strain increases from 0 to 3. Given the previous result on the effect of the length of the orthogonal spring (Fig. 4), we have further analysed the precise role of this other parameter on the equilibrium condition. More specifically, an additional simulation where the deformed length and the residual strain are, respectively, posed equal to 1000 ​nm and 1 (meaning that the initial undeformed length is equal to 500 ​nm) has been performed. In Fig. S3, the two curves representing the retrograde actin flow rate as a function of n_c_, are practically overlapping, and the optimum number of clutches is somewhat identical although the deformed length is not (but is equal to 1000 or 2000 ​nm). This indicating that over a certain value of the orthogonal spring length (500 ​nm), the parameter does not affect the solution and clutch system remains sensitive to elastic strain energy, in turn, dependent on substrate stiffness ($k_{s}=1\;\frac{pN}{nm}$) and solid strain (here 1), introduced in the orthogonal spring.

**Fig. 7:**
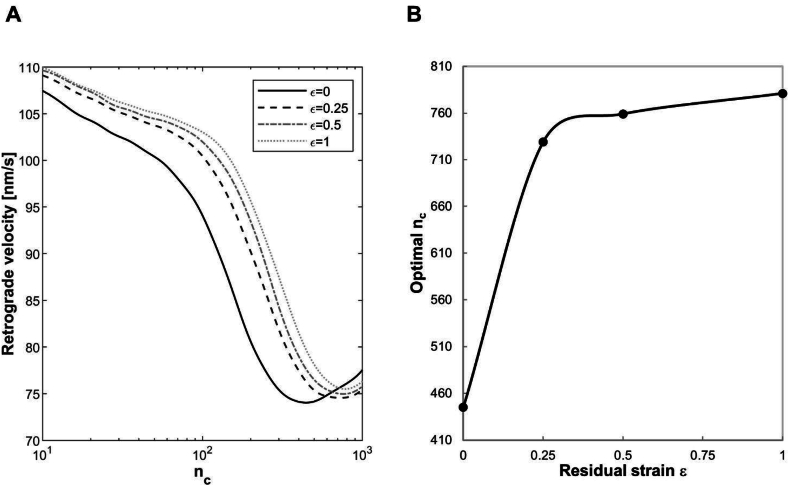
Retrograde actin flow rate as a function of n_c_ predicted by the modified version of motor clutch model when 4 levels of longitudinal residual strain (0, 0.25.0.5 and 1) are introduced in the system (A) and correspondent values of optimal n_c_ (B). Number of Monte Carlo runs equal to 100 ​k, $k_{s}=1\frac{pN}{nm}$.

However, it is interesting to notice that, once introduced the orthogonal spring, a similar trend can be obtained by applying strain/deformation along the direction of the longitudinal one ($k_{s}$ in Fig. 1). In point of fact, the introduced longitudinally residual strain generates again a stiffening effect directly dependent on its level, as evidenced by the shifting of n_c_ towards higher values (Fig. 7). However, the increase of optimal n_c_ is here more relevant than in the case of axial solid strain, resulting equal to 64 ​%, 71 ​% and 76 ​% on the stiff substrate, when solid strain equal to 0.25, 0.5 and 1 are introduced in the orthogonal spring (Fig. 7). The different results associated to axial and transverse residual strain are addressable to the initial geometrical configuration of the orthogonal spring: its rotation, when the solid strain is applied longitudinally on the system, is responsible for the stronger stiffening effect.

The results reported here support our previous findings and confirm cell ability to sense ECM residual strain and to stiffen in response to this specific cue^36^. There is, in fact, a large body of literature that demonstrates the existence of a strict relationship between FA dimension (here represented by the optimal n_c_), traction stresses/forces transmitted to the ECM and CSK mechanical properties [8,41,42]. As previously mentioned, the clutch model in its original version has been implemented to describe cell motility, a cellular function strongly influenced by the ECM properties [43]. Specifically, the original model has provided a mechanistic explanation of stiffness sensitive cell migration and the existence of a stiffness optimum for different cell types [40]. However, although one of the emerging paradigms of mechanobiology is stiffness sensing, there is experimental evidence that cell migration is strongly affected also by deformation and related energy stored inside the tissues. In particular, Márquez et al. have modelled the straintaxis process, a mechanism adopted by cells to move along the direction of increasing strain the elastic substrate [44] and experimentally observed during the embryogenesis process [45]. The cell's ability to sense the surroundings' strain energy and, in particular, residual/solid stresses and to transmit them through its interior as a series of molecular events, has been directly reported in solid tumours. A multitude of biochemical and biophysical factors influence the growth and the migration of tumour cells. Among them, high levels of solid stresses within the tumour revealed a key role in promoting motility of transformed and partially transformed breast cancer cells [46]. If cell migration can be considered an indirect way to demonstrate cell ability to perceive solid/residual stresses, it was clearly demonstrated that solid stress up-regulates the expression of β1 integrin (i.e., n_c_) and other genes in glioblastoma and breast cancer cells [47], and increases intracellular contractility generated by actomyosin cytoskeleton [46].

### 3.4 Threshold and saturation effects for strain energy sensing

**Fig. 8:**
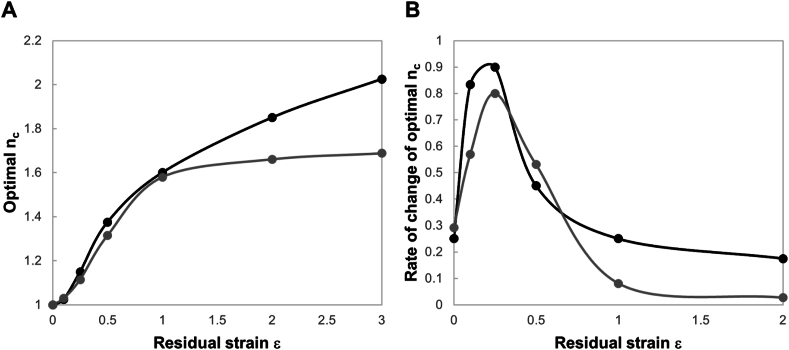
Curves of optimal n_c_ reported in Fig. 6 normalized respect to the optimal n_c_ associated to a null residual strain (40 for $k_{s}=0.1\;\frac{pN}{nm}$ and 445 for $k_{s}=1\;\frac{pN}{nm}$) (A). Rates of changes of normalized optimal n_c_ where the single values (solid points) are evaluated as slopes of the secant lines connecting two consecutive points in A (B). $k_{s}=0.1\frac{pN}{nm}$ (grey curve) and $1\frac{pN}{nm}$ (black curve).

It is interesting to notice that, when the substrate stiffness is equal to $0.1\;\frac{pN}{nm}$, the lowest residual strain considered in our analyses (0.1) induces an increase of the optimal n_c_ by 1, supporting the experimental evidence of a threshold effect for strain energy sensing [39]. In the case of the stiffer substrate, we have not directly found a threshold value for the residual strain, indicating that, if there is, it is lower than that associated with the softer substrate, and suggesting the idea of a greater sensitivity of the clutch system to low residual strain. On the other hand, the analysis of the rates of change of optimal n_c_ indicates the existence of a saturation effect (Fig. 8) similar to that experimentally revealed for stiffness sensing: cells appeared to be no longer able to sense mechanical changes of the microenvironment beyond a certain substrate stiffness [48]. To better compare the curve trends, we have normalised them respect to the optimal n_c_ associated to a null solid strain (40 for $k_{s}=0.1\;\frac{pN}{nm}$ and 445 for $k_{s}=1\;\frac{pN}{nm}$) and evaluated the rates of changes as slopes of the secant lines connecting two consecutive points. For both substrate stiffnesses, the slope increases up to 0.25 (acceleration in the energy sensing process) and then reduces (deceleration): if the acceleration results higher on the soft substrate, the deceleration process proceeds more speedily on the stiff substrate; here, in fact, the rate of change approaches to 0 when the residual strain is equal to 1, indicating that higher residual strain are not able to produce a further and significant increase of the optimal n_c_ (saturation effect). These two effects would set a lower and an upper limit for cell sensing to the strain energy, that are only partially demonstrated and require further experimental validation.

### 3.5 Average solutions of dynamic parameters describing the system

**Fig. 9:**
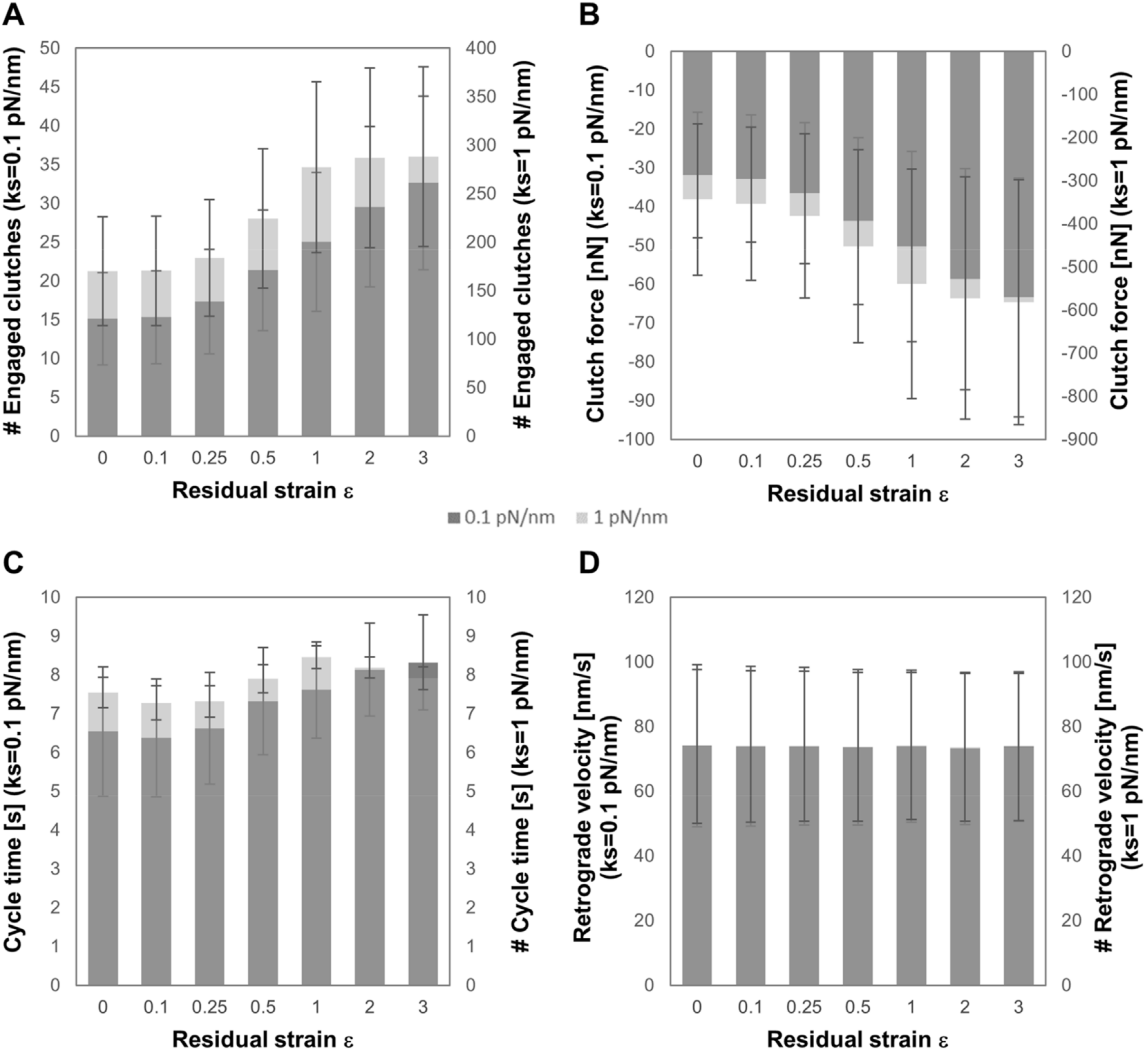
Average values of number of engaged clutches (A), clutch force (B), cycle time (C) and retrograde actin flow rate (D), predicted by the modified version of motor clutch model in correspondence of the optimal n_c_ reported in Fig. 6 for two spring stiffnesses (k_s_ ​= ​0.1, dark grey plot, and 1 ​pN/nm, light grey plot) and 7 levels of residual strain (ε ​= ​0, 0.25, 0.5, 1, 2 and 3). Number of Monte Carlo runs equal to 100 ​k.

Further, single simulations in correspondence of optimal n_c_ have been performed and average outputs (engaged clutches, force generated in the parallel connection of engaged springs, cycle time and retrograde velocity) for axial residual strain and for both substrate stiffnesses are reported in Fig. 9.

In particular, the number of engaged clutches, averaged for all simulated runs, is the index that better reflects the change of FAs length. As shown in Fig. 9, engaged clutches, clutch forces and cycle times increase as substrate stiffness and residual strain increase, as a direct consequence of the stiffening effect already discussed. All these outputs show a threshold effect for both stiffnesses and a saturation effect only on the stiffer substrate, as a consequence of the same trend observed for optimal n_c_ and discussed above. On the contrary, retrograde velocity does not result affected by residual strain and substrate stiffness, but it seems to oscillate stochastically around constant values. A possible option is that the dependence of cycle time on substrate stiffness or energy sensing could be better described by including the tension-dependent strengthening of adhesions, a phenomenon observed experimentally [49,50] and theoretically modelled [51].

## 4 Conclusion

The mechanical behaviour of biological tissues is affected by the presence of the residual stresses. They have been found in blood vessels, heart, bones, trachea, in chick embryos and in solid tumours. It is believed that the residual stress arises to homogenise the transmural stress distribution, altered by growth or remodelling, helping the tissue to optimise its homeostasis and function. They have been reported also in solid tumours, where excessive growth in a confined space may lead to accumulation of stresses, distinct from those generated by fluid components since they are exerted and transmitted through the elastic components of the cancerous tissue^47^.

Here, we have explained the cell response to residual stresses within the same frame of the clutch model introducing a slight modification of the motor clutch to integrate the effect of residual stresses on the strain energy distribution. The results confirmed that cells sense the strain energy stored in the microenvironment, and consequently change their internal mechanical state. This approach could be useful to interpret and understand cell mechanosensing mechanisms and explain cell behaviour during physio-pathological processes, such as embryogenesis, tissue development, wound healing and tumour progression.

## Author's contributions

V.P.: Conceptualization, Methodology, Software, Validation, Formal analysis, Investigation, Writing - original draft, Writing - review & editing, Visualization. C.D.C.: Software, Formal analysis, Investigation, Writing - review & editing, Visualisation; M.R.: Conceptualization, Writing - review & editing; S.F.: Conceptualization, Supervision, Writing - review & editing; P.A.N.: Conceptualization, Resources, Writing - review & editing, Supervision, Project administration, Funding acquisition.

All authors gave final approval for publication and agreed to be held accountable for the work performed therein.

## Funding

We received no funding for this study.

## Declaration of competing interest

The authors declare that they have no known competing financial interests or personal relationships that could have appeared to influence the work reported in this paper.
