# Supplementary material for: Insight to motor clutch model for sensing of ECM residual strain
Source: Mechanobiol Med. 2023 Oct 12;1(2):100025. doi: 10.1016/j.mbm.2023.100025 (PMC12128649; doi:10.1016/j.mbm.2023.100025)
Supplement: Multimedia component 1 [file mmc1.docx]

Supplementary Information

Insight to motor clutch model for sensing of ECM residual strain

*Valeria Panzetta^1,2,3^*, Claudia De Clemente^1,2,3^, Michele Russo^4^, Sabato Fusco^3,5^* and Paolo A. Netti^1,2,3^*

^1^Department of Chemical, Materials and Production Engineering, University of Naples Federico II, 80125 Naples, Italy

^2^Centro di Ricerca Interdipartimentale sui Biomateriali, University of Naples Federico II, 80125 Naples, Italy

^3^Istituto Italiano di Tecnologia, IIT@CRIB, 80126 Naples, Italy

^4^Department of Industrial Engineering, University of Naples Federico II, 80125 Naples, Italy

^5^Department of Medicine and Health Sciences “V. Tiberio”, University of Molise, 86100 Campobasso, Italy

KEYWORDS: Focal Adhesions, Motor Clutch Model, Residual Stress, Elastic Strain Energy.


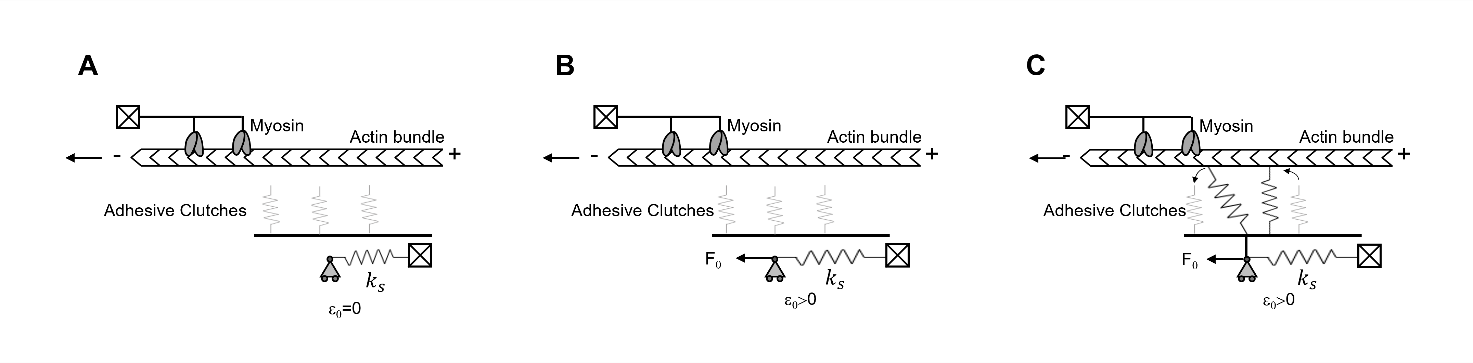


**Figure S1.** Scheme of the original model. Initially, the substrate spring $k_{s}$ is in its rest position (A); then, before any interaction with cell molecular clutches can occur, a force F_0_ is applied to $k_{s}$ which undergoes an axial strain $\varepsilon_{0}$ (B). The adhesive clutches start to engage the substrate exerting an additional force against the ECM and slowing down the actin retrograde flow (C).


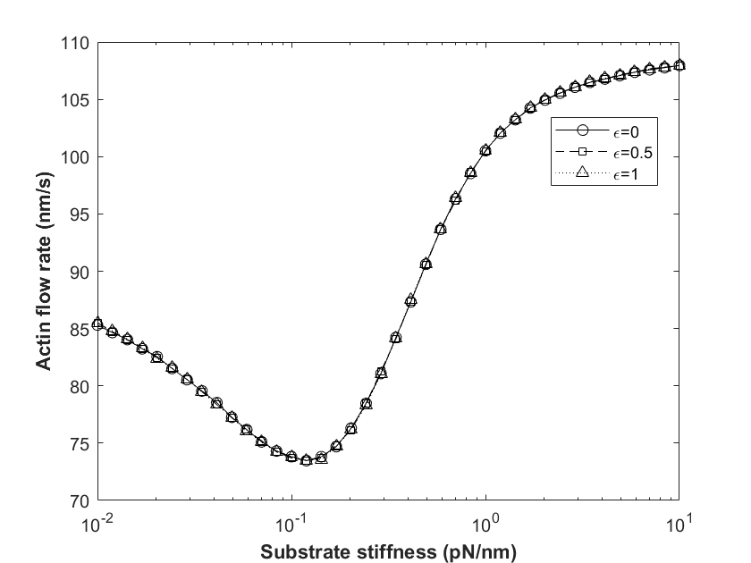

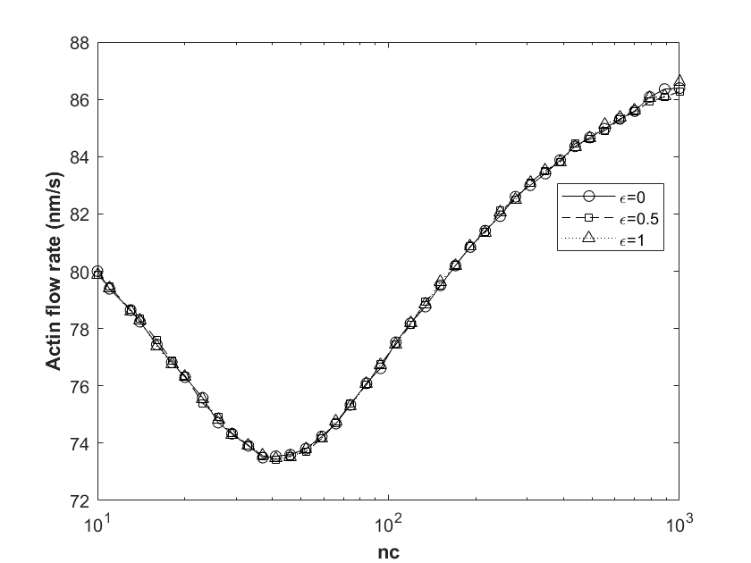


**Figure S2.** Retrograde actin flow rate as a function of substrate stiffness and n_c_ predicted by the original model schematized in the Fig. S1 when 3 levels of residual strain (ԑ=0, 0.5 and 1) are introduced in the system. The optimum substrate stiffness and the optimum number of clutches, that are the minima of the left and right curves, respectively, are not affected by the introduction of the residual strain. This result indicates that the original model predicts that the force transmitted by the molecular clutches to the extracellular environment depends only on the level of the extra strain imposed on top of the already existing residual strain ԑ in the material and not on the strain energy accumulated within the ECM. Number of Monte Carlo runs equal to 1000k.


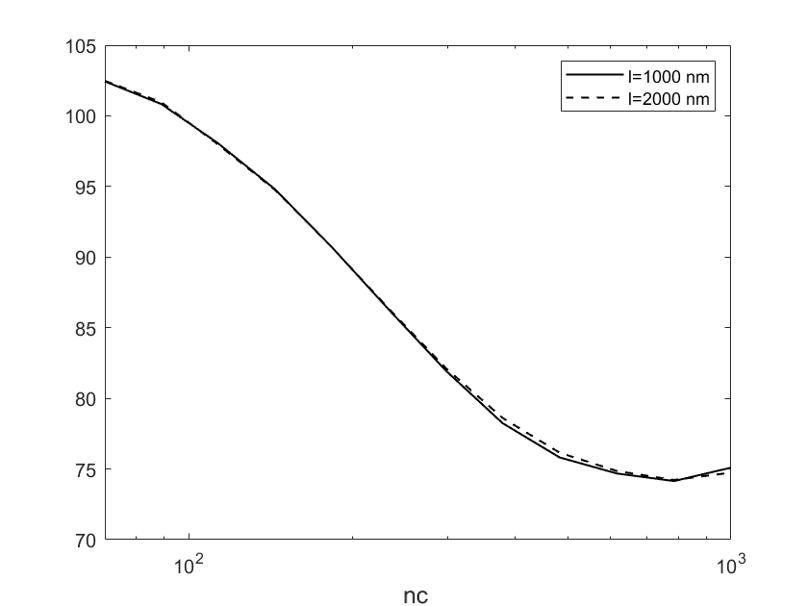


**Figure S3.** Retrograde actin flow rate as a function of n_c_ predicted by the modified version of motor clutch model when the initial length of the orthogonal spring associated to a residual strain equal to 1, is posed equal to 1000 nm (solid line) and to 2000 nm (dashed line). $k_{s}=1\frac{pN}{nm}$ Number of Monte Carlo runs equal to 100k.
